# An Allied Medical Organization

**Published:** 1919-01

**Authors:** 


### An Allied Medical Organization.

The proposition to establish an "inter-allied fellowship of medicine" which will unite more closely American, British, French and other allied schools of medicine, should meet the approval of all physicians of this country. The war has not only brought to the medical profession a more intimate knowledge of medical science, but it has established a relationship among allied physicians that gives to all countries the benefit of the knowledge and experience of other countries—a relationship that by all means should be kept up in the interest of professional advancement.

